# Serum Concentrations of Gastric Intrinsic Factor—A Pilot Study on Patients Undergoing Bariatric Surgery, Presenting with Portal Hypertension, or Suspected Pernicious Anemia

**DOI:** 10.3390/nu18132070

**Published:** 2026-06-24

**Authors:** Eva Greibe, Ebba Nexo, Signe Risgaard Sahlertz, Linda Skibsted Kornerup

**Affiliations:** 1 Department of Clinical Biochemistry, Aarhus University Hospital, 8200 Aarhus, Denmark; enexo@clin.au.dk (E.N.); lindajen@rm.dk (L.S.K.); 2 Department of Clinical Medicine, Aarhus University, 8000 Aarhus, Denmark; 3 Department of Clinical Medicine, Regional Hospital Goedstrup, 7400 Herning, Denmark; sigsah@rm.dk; 4 Department of Hepatology and Gastroenterology, Aarhus University Hospital, 8200 Aarhus, Denmark

**Keywords:** Gastric Intrinsic Factor, ELISA, cobalamin, vitamin B12, retrograde secretion, gastric bypass, portal hypertension, pernicious anemia

## Abstract

**Background/Objectives**: Small quantities of gastric parietal cell-derived Intrinsic Factor (IF) are present in circulation. Here, we explore circulating concentrations of IF in different medical conditions affecting the stomach, anticipating finding both low and high concentrations depending on the state of the parietal cells. **Methods**: We measured serum IF in (I) patients before and after bariatric surgery; (II) patients diagnosed with portal hypertension; and (III) patients with suspected pernicious anemia referred for measurement of IF autoantibodies. **Results**: Patients undergoing bariatric surgery (*n* = 25) showed a significant drop in serum IF following surgery. Their median serum IF declined from 5.1 pmol/L (pre-surgery) to 3.3 pmol/L (two months post-surgery), and nine out of 25 patients showed serum IF concentrations below reference interval (1.7–11.6 pmol/L). Most patients with portal hypertension (*n* = 18) showed normal serum IF concentrations, but with unexpectedly high concentrations up to 30 pmol/L in a few patients. Out of 120 patients referred for measurement of IF autoantibodies, 10 patients showed IF concentrations below the reference interval. Three out of four patients with IF autoantibodies showed low concentrations of circulating IF. **Conclusions**: Our study shows a decline in circulating IF following bariatric surgery consistent with the known decline in vitamin B12 uptake in these patients. Both high and low concentrations were observed in the other two groups. Future longitudinal studies are needed to illuminate possible usefulness for measurement of circulating IF.

## 1. Introduction

Vitamin B12 (cobalamin, B12) is obtained primarily from animal-derived foods such as meat, fish, dairy products, and eggs. It plays a crucial role in DNA synthesis, red blood cell formation, and neurological function. The absorption of B12 is a complex, multi-step process. After ingestion, B12 is released from dietary proteins and subsequently binds to Intrinsic Factor (IF), a glycoprotein secreted by gastric parietal cells. The IF–B12 complex is absorbed in the terminal ileum via receptor-mediated endocytosis. IF is therefore essential for efficient intestinal absorption of B12 [1]. Impaired absorption of B12 may ultimately lead to cellular B12 deficiency, which can be detected by elevated concentrations of methylmalonic acid (MMA), a sensitive functional marker of B12 status [1,2,3]. MMA is therefore commonly used to evaluate the metabolic consequences of reduced B12 uptake. Patients with pernicious anemia have lost the ability to produce IF due to autoimmune atrophic gastritis and are dependent on treatment with pharmacological doses of B12 to prevent deficiency [3]. However, the diagnosis is often delayed to a late stage after symptoms of B12 deficiency have manifested. Previously, diagnosing involved measuring of IF concentrations in gastric juice [4,5], but this cumbersome method requires unpleasant sample collection by nasogastric aspiration (or gastroscopy) and is no longer performed routinely. Another (indirect) approach is to measure autoantibodies developed against IF in blood [6,7]. A positive result confirms pernicious anemia, but a negative result does not rule out the diagnosis or ensure the presence of functional IF [6,7,8].

Small amounts of IF are present in the circulation, presumably reflecting retrograde release from gastric parietal cells. If circulating IF mirrors gastric parietal cell mass or function, alterations might be expected in clinical conditions affecting gastric physiology. Patients undergoing bariatric surgery experience a substantial reduction in functional gastric tissue and are at increased risk of impaired B12 absorption. Patients with pernicious anemia lose IF-producing parietal cells due to autoimmune gastritis [9,10], whereas patients with portal hypertension may develop gastric mucosal abnormalities and parietal cell dysfunction due to the congestive changes caused by the increased pressure [11]. Together, these conditions represent distinct pathophysiological mechanisms that may influence IF production, secretion, or release into the circulation. Because the physiological significance of circulating IF remains poorly understood, we deliberately selected these clinically distinct conditions affecting gastric physiology to explore whether altered circulating IF could be identified.

We previously developed an in-house Enzyme-Linked Immunosorbent Assay (ELISA) for measurement of IF in serum, demonstrating the presence of circulating IF in healthy individuals and establishing a reference interval (1.7–11.6 pmol/L), thereby enabling assessment of IF in blood [12]. Employing this analysis, we report IF levels in patients following bariatric surgery, patients with portal hypertension, and patients with suspected pernicious anemia.

## 2. Materials and Methods

### 2.1. Patients

Serum samples from three different patient groups with suspected disturbances in the gastric function and/or B12 absorption were examined for serum IF: (I) Serum samples from patients undergoing bariatric surgery originated from a previous clinical study investigating the effect of Roux-en-Y gastric bypass (RYBG) and sleeve gastrectomy (SG) on B12 biomarkers [13]. The samples were drawn before and after surgery and analyzed for B12 and B12 biomarkers [13]. For the present study, the serum samples were analyzed for serum IF. (II) Serum samples from patients diagnosed with portal hypertension were collected at two Danish hospitals (Aarhus University Hospital and Regional Hospital Goedstrup) from May 2021 to February 2022. A blood sample was collected at the time of diagnosis, during hospital admission, or in the outpatient clinic. Blood samples were analyzed for serum IF, B12, and MMA. (III) Serum from 120 patients with suspected pernicious anemia referred for measurement of IF autoantibodies at the Department of Clinical Biochemistry, Aarhus University Hospital. For this study, the samples were analyzed for serum IF.

### 2.2. Sample Collection and Analysis

Archival serum samples stored at −80 °C for two to five years were used for patients undergoing bariatric surgery [13], and for approximately one year for patients referred for measurement of IF autoantibodies. Blood samples from patients with portal hypertension (1 × serum tubes 4 mL, 1 × ethylenediaminetetraacetic acid (EDTA) tubes 4 mL, 1 × heparin tubes 4 mL, 1 × sodium citrate tubes 3.5 mL (BD Vacutainer^®^, Lyngby, Denmark)) were collected at Aarhus University Hospital or at Regional Hospital Goedstrup. Routine biochemical analyses were performed at accredited laboratories at Aarhus University Hospital and Regional Hospital Goedstrup (DS/EN ISO/IEC 15189), using comparable analytical methods and reference intervals for hematological parameters and biomarkers for kidney, liver and metabolic functions. All other analytes were analyzed at Aarhus University Hospital. Serum IF was analyzed employing a sandwich ELISA design. The analytical performance characteristics of the assay have previously been described in detail [12], including a limit of detection of 0.19 pmol/L, intra-assay imprecision of approximately 8%, total imprecision of approximately 15%, and documented stability during repeated freeze–thaw cycles and long-term storage. MMA was analyzed on a 6500 Q-TRAP mass spectrometer (Sciex, Copenhagen Denmark) with a High-Performance Liquid Chromatography (HPLC) system (Shimadzu, Kyoto, Japan). Plasma B12 was analyzed on an Atellica Immunoassay (Siemens Healthcare Diagnostics, Ballerup, Denmark). IF autoantibodies were analyzed with an enzymatic EliA method on a Phadia 250 (Thermo Fisher Scientific, Copenhagen, Denmark).

### 2.3. Statistical Analysis

The D’Agostino–Pearson omnibus test was used to determine if the data followed the Gaussian distribution. To compare gastric bypass patients before and after surgery, the paired t-test was used, as the paired differences were normally distributed. To compare the two types of bariatric surgery, the Mann–Whitney U test was used, as data was not normally distributed. Intra-individual coefficient of variation, CV%, for the bariatric patients was calculated based on two samples collected pre-operation. Values of *p* ≤ 0.05 were accepted as statistically significant. The data analysis was performed using the statistical software available in GraphPad Prism version 8.0.1.

## 3. Results

We studied the concentration of IF in the circulation of three groups of patients with different expected disturbances in the gastric function: 25 patients undergoing bariatric surgery, 18 patients with portal hypertension, and 120 patients referred for measurement of IF autoantibodies. Table 1 displays age distribution and preoperative measures of B12-related biomarkers for patients undergoing bariatric surgery and patients diagnosed with portal hypertension. No biomarker values were available for the patients referred for measurement of IF autoantibodies, except for the results on the autoantibodies.

Most patients undergoing bariatric surgery had a normal B12 status prior to surgery, with MMA values largely within the reference interval (Table 1). However, as previously reported [13], MMA levels increased two months after surgery, indicating early metabolic signs of B12 deficiency. Changes in B12 and MMA following bariatric surgery, including comparisons between RYGB and SG, have previously been described in detail [13]. Due to the limited sample size, particularly in the SG group, these data were not stratified in the present study. For patients with portal hypertension, median serum B12 and MMA were within reference intervals (Table 1).

**Figure 1:**
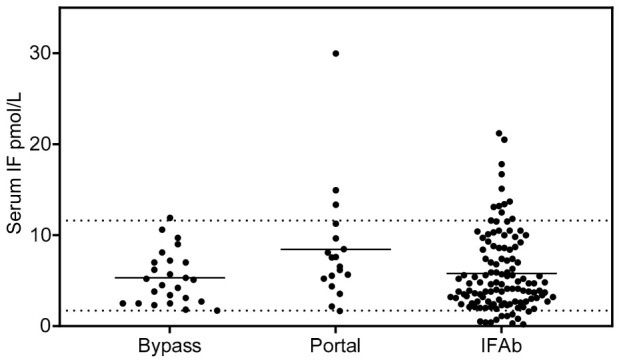
Serum Intrinsic Factor (IF) in three patient groups. Bypass: Patients with obesity before gastric bypass surgery (*n* = 25). Portal: Patients diagnosed with portal hypertension (*n* = 18). IFAb: Patients referred for measurement of IF autoantibodies (*n* = 120). For each group of patients, the individual observations are indicated with dots, and the mean concentration is indicated with a black horisontal line. The 95% reference interval from [12] is indicated with dotted lines.

Serum IF levels for all patient groups are displayed in Figure 1. The reference interval for serum IF in healthy individuals (1.7–11.6 pmol/L) as previously established [12] is indicated as dotted lines in the figure.

**Figure 2:**
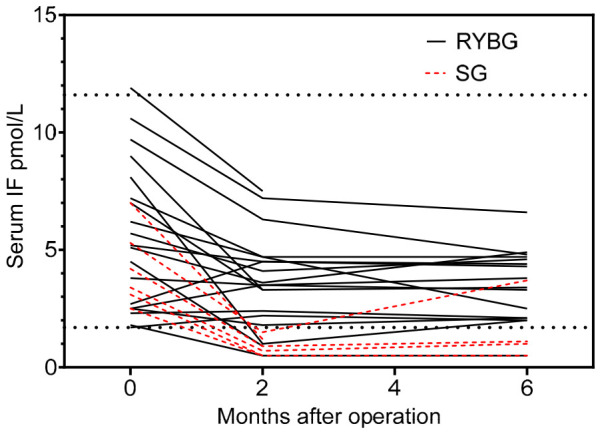
Serum Intrinsic Factor (IF) in patients undergoing gastric bypass. Serum samples were removed before surgery (0 months) and 2 and 6 months after surgery. Patients had either sleeve gastrectomy (SG) (*n* = 6) or Roux-en-Y Gastric Bypass (RYGB) (*n* = 19). The samples were derived from [13]. The 95% reference interval from [12] is indicated with dotted lines.

For the 25 patients undergoing bariatric surgery, serum IF was measured twice prior to surgery (collected within a median of 17 weeks prior to surgery) showing values with an intra-individual variation of <10%, which is consistent with the low intra-individual variation in serum IF concentrations previously observed in healthy individuals [12]. Median serum IF showed a decline from 5.1 [range 1.7–11.9] pmol/L before surgery to 3.3 [range 0.5–7.5] pmol/L two months after surgery (*p* < 0.0001). At this point, nine out of 25 patients had serum IF concentrations below the reference interval. In a descriptive subgroup analysis, the decline appeared numerically greater in SG patients (*n* = 6) than in RYGB patients (*n* = 19) (3.0 pmol/L versus 1.5 pmol/L, respectively). However, this difference did not reach statistical significance (*p* = 0.133), and the comparison is limited by the small number of SG patients. Serum IF concentrations remained low six months after surgery (Figure 2).

The 18 patients with portal hypertension had median serum IF concentrations of 7.0 [range 1.7–30.0] pmol/L. Serum IF concentrations above the reference interval were observed in three patients, including one with an unexpectedly high concentration of 30 pmol/L (Figure 1).

**Figure 3:**
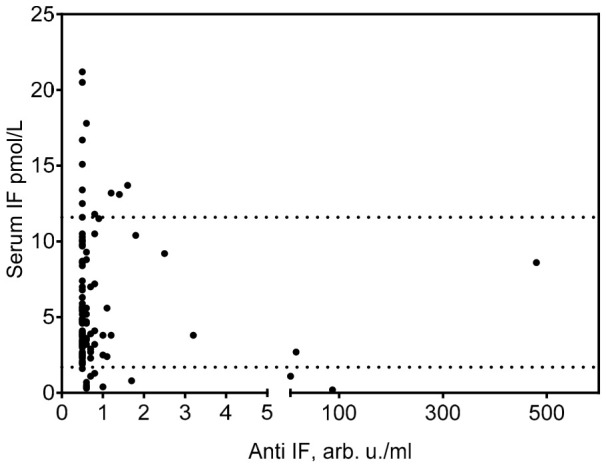
Serum Intrinsic Factor (IF) in 120 patients referred for measurement of autoantibodies against IF in the routine setting at Aarhus University Hospital, Denmark. The 95% reference interval for serum IF from [12] is indicated with dotted lines.

Out of 120 patients referred to measurement of IF autoantibodies, 10 patients had IF concentrations below the reference interval, while 11 patients displayed concentrations above the reference interval (Figure 1). Figure 3 shows serum IF as a function of the results for autoantibodies against IF. Four patients tested positive for IF autoantibodies, consistent with pernicious anemia, and three out of these patients showed low concentrations of circulating IF.

## 4. Discussion

IF is a glycoprotein synthesized by the parietal cells of the stomach and is essential for the intestinal uptake of B12. We have previously documented the presence of minute amounts of IF in human serum [12]. The biological origin and physiological significance of circulating IF remain uncertain and may reflect a combination of parietal cell mass, IF production, secretion dynamics, and retrograde release into the circulation. This raises the question of whether circulating IF could provide information on gastric physiology, meriting further investigation as a biomarker. The current study therefore aimed to explore serum IF concentrations in clinical conditions expected to affect gastric physiology.

In support of this hypothesis, we observed a significant decline in serum IF in bariatric patients two and six months after surgery compared with pre-surgery values. Both RYGB and SG were associated with reduced serum IF concentrations after surgery, supporting the notion that bariatric surgery affects gastric IF production and/or secretion. Although the observed decline in serum IF appeared greater in SG than in RYGB, this subgroup comparison was exploratory and limited by the small number of SG patients, and no procedure-specific conclusions can be drawn. The observed decline in serum IF likely reflects reduced parietal cell function following surgery, which is supported by the concomitant increase in MMA, as previously reported [13]. Bariatric surgery is known to affect B12 metabolism through several physiological and anatomical mechanisms [14,15,16]. Reduced gastric acid secretion following surgery impairs the release of B12 from dietary proteins, which is a prerequisite for its subsequent binding to IF. In addition, the limited parietal cell mass in the remaining functional stomach, particularly in RYGB, leads to decreased production of IF. The altered gastrointestinal anatomy further limits the interaction between IF and B12, as well as its absorption in the terminal ileum. Together, these mechanisms contribute to the increased risk of B12 deficiency observed after bariatric surgery. In this context, the decline in circulating IF reported in the present study likely reflects both reduced parietal cell mass and altered gastric physiology, supporting the potential of serum IF as a marker of impaired gastric function following bariatric procedures. Patients undergoing bariatric surgery are known to experience substantial postoperative changes in dietary intake, including reduced food consumption and altered nutrient composition [17,18]. Although such changes may contribute to alterations in B12 status, they are unlikely to directly influence circulating IF concentrations. In addition, our previous findings of no diurnal variation in circulating IF suggest that the concentration is not affected by intake of food [12].

Prior to the study, we hypothesized that portal hypertension might increase circulating IF concentrations through mucosal congestion, altered gastric mucosal integrity, and enhanced leakage of IF into the circulation. However, we observed median serum IF concentrations within the reference interval. The portal hypertension cohort comprised patients with different underlying etiologies and varying degrees of liver dysfunction. These factors may have influenced both B12-related biomarkers and circulating IF concentrations and could therefore have contributed to the variability observed within this patient group. The function of the gastric mucosa has been shown to be affected in patients with portal hypertension causing parietal cell dysfunction and even loss of parietal cell mass. Consequently, gastrin levels increase because of the simultaneous decrease in gastric acid excretion [19,20]. Gastrin may stimulate IF production in the remaining parietal cells, potentially explaining the observed IF levels within the normal range. In addition, treatment with beta-blockers, commonly used in patients with portal hypertension to reduce portal pressure [21], may influence gastric mucosal perfusion and thereby indirectly affect parietal cell function. This could potentially modulate IF secretion and contribute to the variability in circulating IF concentrations observed in this group. However, this remains speculative since medication data were not available in the present study. Overall, our findings do not support a consistent increase in circulating IF in patients with portal hypertension, although elevated serum IF concentrations were observed in a few individuals.

Finally, we measured serum IF in patients suspected of suffering from pernicious anemia and referred for measurement of IF autoantibodies in a routine setting. Because this cohort represented patients referred for IF autoantibody testing rather than patients with confirmed pernicious anemia, considerable heterogeneity was expected. Nevertheless, we anticipated that some patients would display reduced circulating IF concentrations. Low IF concentrations were observed in 10 patients, whereas 11 patients had elevated concentrations. The observed variability cannot be readily interpreted because clinical information, final diagnoses, and complementary biomarkers were unavailable. Consequently, the relationship between circulating IF, IF autoantibody status, and underlying gastric pathology remains uncertain.

The present study has several strengths. First, we applied a newly developed and analytically validated in-house ELISA, enabling direct measurement of circulating IF, which has previously been difficult to assess in clinical practice. Second, we included three clinically distinct patient groups with different expected alterations in gastric physiology, allowing exploration of circulating IF across a range of clinically relevant conditions. Third, the longitudinal design in the bariatric cohort, with repeated pre- and postoperative measurements, strengthens the observed association between reduced serum IF and impaired B12 metabolism. Importantly, this study is, to our knowledge, the first to assess circulating IF across different clinical conditions using a validated serum-based method. At the same time, the present study has several limitations. The sample size was relatively small, and the patient groups were heterogeneous, representing different clinical conditions with distinct underlying pathophysiological mechanisms. Consequently, the study was not designed to identify disease-specific patterns of circulating IF or to establish a common biological mechanism across the investigated conditions. Rather, the findings should be interpreted as hypothesis-generating exploratory observations regarding the relationship between circulating IF and gastric function. In particular, the portal hypertension cohort lacked longitudinal follow-up, limiting the ability to assess changes in serum IF over time in relation to disease progression. In addition, B12-related biomarkers were not available for the group of patients referred for IF autoantibody testing, and correlations between these biomarkers and serum IF could therefore not be assessed. Information on medication use and dietary habits was not available across the study populations. Such factors, including restrictive diets and treatments such as metformin, may influence B12 status and could potentially contribute to variation in circulating IF concentrations. The lack of consistent data on clinical variables such as alcohol consumption, medication use, and comorbidities is a limitation of the present study. Taken together, the findings should be regarded as exploratory and hypothesis-generating. While the observed decline in circulating IF following bariatric surgery supports a relationship between serum IF and altered gastric physiology, the biological significance and potential clinical utility of circulating IF remain to be established in larger, well-characterized longitudinal studies.

## 5. Conclusions

In conclusion, we found reduced serum IF following bariatric surgery, consistent with the known risk of B12 deficiency in these patients. The findings in patients with portal hypertension and suspected pernicious anemia remain heterogeneous. Because the investigated patient groups were clinically heterogeneous, the findings should be regarded as exploratory and hypothesis-generating rather than disease-specific. Longitudinal studies with detailed clinical characterization and comprehensive assessment of B12-related biomarkers are needed to clarify the potential clinical utility of circulating IF.

## Figures and Tables

**Table 1:** Age and B12-related biomarkers prior to bariatric surgery and at inclusion for the portal hypertension cohort ^1^.

	Reference Interval ^2^	Bariatric Surgery Patients (Preoperative) ^3^ *n* = 25 (67% Females)	Portal Hypertension Patients *n* = 18 (22% Females)
Age, years		43 [26–65]	58 [21–75]
B12, pmol/L	>250	296 [178–554]	671 [283–1253]
MMA, µmol/L	0.10–0.40	0.18 [0.12–0.41]	0.18 [0.06–1.47]

^1^ Age and B12-related biomarkers in patients undergoing bariatric surgery (preoperative measurements) and in patients with portal hypertension at time of inclusion. Age and biomarker values were not available for patients referred for measurement of IF autoantibodies. Results are presented as medians with [range]. ^2^ Reference intervals are from the routine laboratory at Aarhus University Hospital, Denmark. ^3^ Data originate from [13].

## Data Availability

The Danish Data Protection and the European act on General Data Protection Regulation do not allow for personal data to be made available to other researchers without prior written approval from the relevant institutions and authorities. For further information, please contact the corresponding author.

## Abbreviations

The following abbreviations are used in this manuscript:

IF	Gastric Intrinsic Factor
B12	Vitamin B12
ELISA	Enzyme-Linked Immunosorbent Assay
EDTA	Ethylenediaminetetraacetic Acid
HPLC	High-Performance Liquid Chromatography
RYBG	Roux-en-Y Gastric Bypass
SG	Sleeve Gastrectomy
MMA	Methylmalonic Acid
IFAb	Intrinsic Factor Autoantibodies
CV%	Coefficient of Variation
